# What’s the Function of Connexin 32 in the Peripheral Nervous System?

**DOI:** 10.3389/fnmol.2018.00227

**Published:** 2018-07-10

**Authors:** Mario Bortolozzi

**Affiliations:** ^1^Department of Physics and Astronomy G. Galilei, University of Padua, Padua, Italy; ^2^Venetian Institute of Molecular Medicine (VIMM), Padua, Italy; ^3^Padova Neuroscience Center (PNC), Padua, Italy

**Keywords:** Cx32, Connexin 32, Schwann cell, CMT1X, CMTX1, Charcot-Marie-Tooth disease, gap junction, hemichannel

## Abstract

Connexin 32 (Cx32) is a fundamental protein in the peripheral nervous system (PNS) as its mutations cause the X-linked form of Charcot–Marie–Tooth disease (CMT1X), the second most common form of hereditary motor and sensory neuropathy and a demyelinating disease for which there is no effective therapy. Since mutations of the *GJB1* gene encoding Cx32 were first reported in 1993, over 450 different mutations associated with CMT1X including missense, frameshift, deletion and non-sense ones have been identified. Despite the availability of a sizable number of studies focusing on normal and mutated Cx32 channel properties, the crucial role played by Cx32 in the PNS has not yet been elucidated, as well as the molecular pathogenesis of CMT1X. Is Cx32 fundamental during a particular phase of Schwann cell (SC) life? Are Cx32 paired (gap junction, GJ) channels in myelinated SCs important for peripheral nerve homeostasis? The attractive hypothesis that short coupling of adjacent myelin layers by Cx32 GJs is required for efficient diffusion of K^+^ and signaling molecules is still debated, while a growing body of evidence is supporting other possible functions of Cx32 in the PNS, mainly related to Cx32 unpaired channels (hemichannels), which could be involved in a purinergic-dependent pathway controlling myelination. Here we review the intriguing puzzle of findings about Cx32 function and dysfunction, discussing possible directions for future investigation.

## Introduction

Connexin 32 (Cx32) is a 32 kDa protein of the connexin family, abundantly found in liver (Paul, 1986), but it is also expressed in many other tissues, including the central nervous system (CNS) and the peripheral nervous system (PNS) (Scherer et al., 1995; Rash et al., 2001). Mutations in the *GJB1* gene, which encodes Cx32, are the leading cause of the X-linked dominant form of Charcot–Marie–Tooth disease (CMT1X or CMTX1), the second most common form of hereditary motor and sensory neuropathy and a disease for which there is no cure (Kleopa and Scherer, 2006; Kleopa et al., 2012). Since mutations were first reported in 1993 (Bergoffen et al., 1993), over 450 different mutations associated with CMT1X including missense, frameshift, deletion and non-sense ones have been identified according to The Human Gene Mutation Database (HGMD^®^; Stenson et al., 2014). In both nerves and ganglia of the PNS, Cx32 localizes only in myelinating Schwann cells (SCs), mainly to the paranodes, the periodic interruptions in the compact myelin called Schmidt–Lanterman incisures, and the two outer layers of myelin (Scherer et al., 1995; Meier et al., 2004; Procacci et al., 2008). Elucidation of the molecular function of Cx32 in myelinating SCs is a requirement for understanding how different mutations lead to the sequence of events that end in demyelination and axonal loss in CMT1X patients. Despite the availability of an incredible number of studies, mostly *in vitro*, focusing on normal and mutated Cx32 channel properties, an interpretative framework is still lacking.

### Cx32 Gap Junction Channels in Non-compact Regions of Myelinating Schwann Cells

Minute intracellular gap junction (GJ) channels are formed in non-compact regions of SC myelin when hexamers of Cx32 (connexons or hemichannels) spanning opposite myelin layers dock end-to-end (Meier et al., 2004), providing a fast diffusive radial path between the abaxonal and adaxonal region (Balice-Gordon et al., 1998). This has led to the attractive hypothesis that Cx32 GJ channels are critical for the passage of K^+^ and signaling molecules across the myelin sheath of SCs, whose function is not only to myelinate axons but also to maintain their long-term functional integrity (Nave and Trapp, 2008). The overlap in the distribution of Cx32 and Cx29 at incisures and paranodes suggests that they both contribute to reflexive junctions (Altevogt et al., 2002; Li et al., 2002), even if immunogold labeling for Cx29 did not reveal ultrastructurally defined GJs at incisures or directly linking successive paranodal loops (Li et al., 2002). Furthermore Cx29 does not form junctional channels when expressed in cultured cells (Altevogt et al., 2002; Ahn et al., 2008), neither Cx29 human ortholog Cx31.3 (Sargiannidou et al., 2008). Cx43 is also highly expressed in paranodal regions of myelinating SCs of adult wild-type (WT) and Cx32-null mice (Zhao et al., 1999). In adult rat, staining of Cx43 along myelin sheath and SC bodies was observed, but lower than Cx32 (Mambetisaeva et al., 1999), whereas an immunohistochemical study of human peripheral nerves revealed that anti-Cx43 antibody stained cytoplasm around the nucleus of SCs but not myelin (Yoshimura et al., 1996). Cx26 is also expressed in myelinating SCs of neonatal WT mice (Zhao et al., 1999), but not in those of neonatal rats (Mambetisaeva et al., 1999), supporting the conclusion that connexin expression in SCs is, at least in part, species-dependent, so direct investigation in the human nerve would be needed.

### Cx32 Hemichannels May Participate in the Myelination Process of Schwann Cells

In the PNS, electrical stimulation of myelinated nerves triggers axonal ATP release which induces Ca^2+^ increases in the cytosol and the mitochondrial matrix of the surrounding SCs via P2Y receptor activation (Lev-Ram and Ellisman, 1995; Lyons et al., 1995; Mayer et al., 1997; Stevens and Fields, 2000; Ino et al., 2015). This neuron-to-SC pathway is likely to have an important role in proper myelination as *in vivo* chronic suppression of the purinergic-mediated signaling inhibits correct myelin formation and causes hypomyelination (Ino et al., 2015). Cx32 hemichannels in myelinating SCs may contribute to regulate the myelination process by enhancing the intracellular and intercellular propagation of this Ca^2+^ signaling by a regenerative ATP-induced ATP release mechanism. The presence of functional Cx32 hemichannels was recently hypothesized based on connexin-mediated ATP release observed during electrical stimulation of mice sciatic nerves (Nualart-Marti et al., 2013). Indeed, the molecular machinery ideally suited to support a Cx32-mediated purinergic signaling throughout SCs is actually present in peripheral nerves, given that Cx32, IP_3_R and P2Y receptors are found together in the paranodes and in the outer layer of SCs (Martínez-Gómez and Dent, 2007; Toews et al., 2007) and Cx32 hemichannels can release ATP (Cotrina et al., 2000; Belliveau et al., 2006; De Vuyst et al., 2006; Nualart-Marti et al., 2013). Indeed, comparing SCs cultured from sciatic nerves of WT and Cx32-null mice, Cx32 was found to enhance the intercellular Ca^2+^ waves spreading without contribution of Cx32 GJs (Zhao et al., 1999). As the Ca^2+^ wave propagation was mediated by extracellular release of ATP, it can reasonably be inferred the involvement of Cx32 hemichannels. The same consideration applies to another work (Freidin et al., 2009) using primary cultures of purified SCs from sciatic nerve which suggests a link between Cx32 expression and GGF2 (a growth factor which controls SC proliferation and differentiation), which does not involve Cx32-mediated GJ communication.

### Other Possible Functions of Cx32 in Myelinating Schwann Cells

*GJB1* gene depletion results in a mitotic phenotype from the genome-wide phenotypic profiling performed by the Mitocheck consortium (Neumann et al., 2010). Mitotic instability and CMT1X phenotype were linked to increased CaMKII activity in both human and murine fibroblasts carrying the G12S and S26L mutations of Cx32 as normal mitosis and motor function of mutant mice were partially recovered by CaMKII inhibitors (Mones et al., 2012, 2014). Cx32-S26L hemichannel dysfunction due to altered CaMKII activity was also proposed (Mones et al., 2014), supporting the notion that a CaM-dependent pathway controls the hemichannel gating by cytosolic Ca^2+^ of α and β connexin isoforms (De Vuyst et al., 2006, 2009; Zhang et al., 2006; Zhou et al., 2009; Hu et al., 2018). As found in oligodendrocytes (Waggener et al., 2013), CaMKII may be also critical in SCs for the well balanced equilibrium between dynamic remodeling and kinetic stability of the actin cytoskeleton required for efficient myelination.

Recently (Fowler et al., 2013), by employing a proteomic approach in murine liver, it has been reported that Cx32 is expressed in the inner mitochondrial membrane and interacts with the outer mitochondrial membrane resident fraction of syderoflexin-1 (SFXN-1), thus suggesting a putative role for Cx32-SFXN1 axis as protein complex for mitochondrial plasma membrane tethering. In Cx32-null mice, several mitochondrial proteins are upregulated, indicating that Cx32 optimizes mitochondrial bioenergetics by restricting rates of oxidative phosphorylation (Fowler et al., 2017, communication at the International GJ Conference).

### The X-Linked Form of Charcot-Marie-Tooth Disease

CMT1X patients develop progressive distal muscle weakness and amyotrophy, together with sensory abnormalities that are most pronounced in the distal extremities (Vance, 1991; Harding, 1995; Suter and Snipes, 1995). CNS disturbances, mainly episodic but in some cases including static deafness and cognitive impairment, can occur in CMT1X patients, whereas the symptoms do not appear to correlate with the stage and severity of the peripheral neuropathy (Abrams and Freidin, 2015; Wang and Yin, 2016). Nerve electrophysiological and pathological analysis show intermediate slowing of conduction and length-dependent axonal loss, with more prominent axonal degeneration than de/remyelination (Kleopa et al., 2012), which supports the hypothesis that axonal abnormalities precede demyelination (Vavlitou et al., 2010). Interestingly, these studies together show that clinical and pathophysiological features of patients lacking the entire coding region of Cx32 are similar to that of most other patients with CMT1X, suggesting that most mutations cause loss-of-function (Shy et al., 2007; Kleopa, 2011). Functional analysis of Cx32 mutations (mainly CMT1X-related) using various expression systems has revealed a plethora of alterations that could schematically subdivided in classes, not mutually exclusive, which range from mutations that cause loss of channel formation to those that retain electrical coupling but show altered permeation properties or defective gating mechanisms:

1. Cx32 protein is not synthesized (Ionasescu et al., 1996; Ainsworth et al., 1998; Abrams and Freidin, 2015).
2. Mutant Cx32 protein is normally transcribed but little protein is expressed in the cell, such as in a frameshift of Cx32 at amino acid 175 (Deschenes et al., 1997). Some *GJB1* gene mutations in non-coding regions controlling Cx32 expression should belong to this category (Tomaselli et al., 2017).
3. Mutant Cx32 protein is properly synthesized but not transported to the plasma membrane, causing abnormal and possibly toxic accumulation in intracellular compartment such as the endoplasmic reticulum (ER), the Golgi apparatus or the cytoplasm. This condition results for mutations W3D (Kalmatsky et al., 2012), W3Y (Martin et al., 2000), Y7D (Kalmatsky et al., 2012), G12S (Deschenes et al., 1997; Wang et al., 2004; Kalmatsky et al., 2009; Mones et al., 2014), R32E (Fleishman et al., 2006), M34K (Yum et al., 2002), M34T (Yum et al., 2002), V38M (Yum et al., 2002), A39P and A39V (Kleopa et al., 2002), A40V (Yum et al., 2002), F51L (Abrams et al., 2017), C53S (Yoshimura et al., 1998), T55I (Kleopa et al., 2002; Sargiannidou et al., 2009; Abrams et al., 2017), R75P and R75Q (Yum et al., 2002), R75W (Yum et al., 2002; Sargiannidou et al., 2009; Abrams et al., 2013), E102del (Abrams et al., 2017), V140E (Kleopa et al., 2006), R142E (Fleishman et al., 2006), R142Q (Abrams et al., 2017), R142W (Deschenes et al., 1997; Vanslyke et al., 2000; Jeng et al., 2006; Abrams et al., 2017), L143P (Kleopa et al., 2006), E146R (Fleishman et al., 2006), R164Q (Kleopa et al., 2002; Abrams et al., 2017), R164W (Kleopa et al., 2002), C168Y (Abrams et al., 2017), P172R (Yoshimura et al., 1998), V177A (Abrams et al., 2017), E186K (Deschenes et al., 1997; Vanslyke et al., 2000), N205I (Yum et al., 2002), E208K (Deschenes et al., 1997; Martin et al., 2000; Vanslyke et al., 2000; Wang et al., 2004), Y211X and C217X (Yum et al., 2002).
4. Mutant Cx32 protein is transported and inserted in the membrane to form hemichannels but no plaques or functional GJ channels are found. This condition results for mutations W3S (Martin et al., 2000), R15W (Abrams et al., 2001), R22G and R22P (Ressot et al., 1998), C60F (Omori et al., 1996), R75D-R75E-R75P-R75Q-R75V (Abrams et al., 2013), L90H (Ressot et al., 1998), H94Y (Abrams et al., 2001), V95M (Ressot et al., 1998), V139M (Omori et al., 1996; Deschenes et al., 1997; Abrams et al., 2001, 2017), R142Q and R164W (Abrams et al., 2017), P172S (Ressot et al., 1998), N175Y (Nakagawa et al., 2011), S182T (Wang et al., 2004), E208L (Ressot et al., 1998), E208K (Castro et al., 1999), Y211X (Ressot et al., 1998; Castro et al., 1999; Wang et al., 2004), I214X (Rabadan-Diehl et al., 1994), R215Q (Castro et al., 1999), R215W (Omori et al., 1996; Castro et al., 1999), R215X (Rabadan-Diehl et al., 1994; Castro et al., 1999), C217X (Rabadan-Diehl et al., 1994).
5. Mutant Cx32 protein forms electrically conductive GJ channels and hemichannels presenting with altered properties in respect to WT channels, e.g., decreased/increased channel number, distribution, gating sensitivity or unitary permeability to physiologically crucial ions and molecules. Studies of functionality refers to mutations N2A-N2D-N2E-N2Q (Oh et al., 1999), G12S (Abrams et al., 2001), V13L (Martin et al., 2000; Wang et al., 2004), R15Q (Abrams et al., 2001; Wang et al., 2004), R22Q (Wang et al., 2004), S26L (Oh et al., 1997; Bicego et al., 2006; Mones et al., 2014), I30N (Oh et al., 1997; Wang et al., 2004), M34T (Oh et al., 1997), V35M (Oh et al., 1997; Wang et al., 2004), V38M (Oh et al., 1997), S42E (Oh et al., 1999), L56F (Ressot et al., 1998), V63I (Wang et al., 2004), Y65C (Wang et al., 2004), R75A-R75H-R75K-R75L (Abrams et al., 2013), R75Q (Wang et al., 2004), Q80R (Wang et al., 2004), S85C (Abrams et al., 2001), P87A (Oh et al., 1997), H94Q (Abrams et al., 2001), V95M (Wang et al., 2004), E102G (Oh et al., 1997; Ressot et al., 1998; Abrams et al., 2003), R107W (Wang et al., 2004), Del 111–116 (Oh et al., 1997; Ressot et al., 1998; Bicego et al., 2006), W133R (Wang et al., 2004), Y151C (Abrams et al., 2017), L156R (Wang et al., 2004), P158A-R164W-P172S (Wang et al., 2004), N175D (Gong et al., 2013), V181A (Abrams et al., 2003), V181M and R183C (Abrams et al., 2017), G199R (Wang et al., 2004), N205S (Kleopa et al., 2002; Wang et al., 2004), R215X (Rabadan-Diehl et al., 1994), C217X (Rabadan-Diehl et al., 1994; Castro et al., 1999), R220X (Rabadan-Diehl et al., 1994; Omori et al., 1996; Deschenes et al., 1997; Ressot et al., 1998; Castro et al., 1999; Revilla et al., 1999; Bicego et al., 2006; Katoch et al., 2015; Carrer et al., 2018), R223X and N226X (Rabadan-Diehl et al., 1994), R238H (Castro et al., 1999), L239I (Abrams et al., 2017), C280G (Castro et al., 1999; Kleopa et al., 2002), S281X (Castro et al., 1999).

Most of these studies were limited to testing only GJ channel electrical conductance, so minimal information is available about specific permeability to important molecules up to 1 kDa (e.g., second messengers), which could explain why some mutants appear as “functional” with respect to the WT. Limited information is also available about gating/permeability dysfunction of mutant Cx32 hemichannels, analyzed in the following studies: S26L (Mones et al., 2014), S85C (Abrams et al., 2002), D178Y (Gómez-Hernández et al., 2003), E208K-Y211X-R215X-R215W-R215Q-C217X (Castro et al., 1999), R220X (Castro et al., 1999; Carrer et al., 2018), F235C (Liang et al., 2005), R238H-R265X-C280G-S281X (Castro et al., 1999).

**Figure 1:**
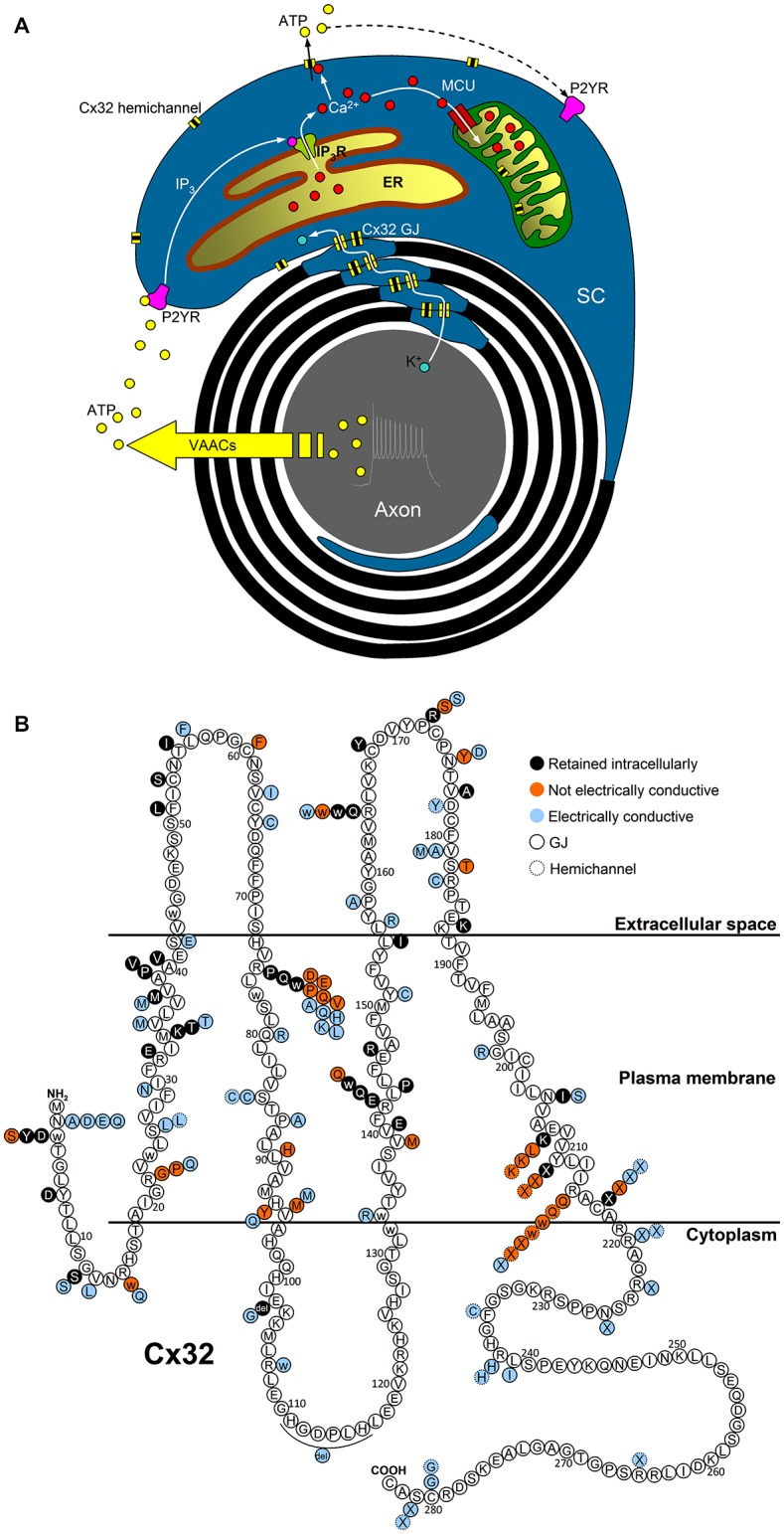
Possible functions of Cx32 in myelinating SCs and topology of Cx32 mutations. **(A)** Electrical activity of myelinated nerves triggers axonal K^+^ release, whose recycling could involve Cx32 GJs located in SC paranodes and Schmidt–Lanterman incisures. Axonal firing also stimulates ATP release from volume-activated anion channels (VAACs; Fields and Ni, 2010) which induces P2Y-mediated Ca^2+^ increases in the cytosol and the mitochondrial matrix of the surrounding SCs via IP_3_ receptors (IP_3_R) of the endoplasmic reticulum (ER) and the mitochondrial calcium uniporter (MCU), respectively. The increase in the cytosolic Ca^2+^ concentration ([Ca^2+^]_i_) should be sufficient to trigger Cx32 hemichannel opening and ATP release, contributing to the intracellular and intercellular propagation of the Ca^2+^ signal. Interaction between Cx32 hemichannels and mitochondria may play a role in cell bioenergetics as found in liver of Cx32-null mice (Fowler et al., 2013). **(B)** Cx32 mutations belonging to classes 3-4-5 mentioned in the text are represented as colored circles (black-red-azure, respectively) associated to the correspondent WT amino acid (white circle), where the topology of Cx32 domains is derived from the all-atom model of Cx32 connexon in Carrer et al. (2018).

Figure 1 outlines a graphical summary of: (i) possible functions of Cx32 in myelinating SCs; and (ii) the available information about *in vitro* functional studies of Cx32 mutations. Interestingly, the expression and function of some Cx32 mutants are cell-dependent, e.g., mutants R75Q, M34T, V38M, R164W, Y211X, C217X that reach the plasma membrane in non-human non-glial *Xenopus oocytes* and N2A cells (Oh et al., 1997; Castro et al., 1999; Wang et al., 2004), fail to reach the plasma membrane in human cells (HeLa) and cultured rat SCs (Kleopa et al., 2002; Yum et al., 2002).

Together with the Cx32-null mouse (Nelles et al., 1996; Anzini et al., 1997; Balice-Gordon et al., 1998; Scherer et al., 1998, 2005; Zhao et al., 1999; Nicholson et al., 2001; Freidin et al., 2015; Sargiannidou et al., 2015; Kagiava et al., 2016), which displays a progressive peripheral neuropathy, a limited number of CMT1X mice models has been developed, including mutations G12S (Mones et al., 2012, 2014), S26L (Mones et al., 2012, 2014), T55I (Sargiannidou et al., 2009), R75W (Sargiannidou et al., 2009), R142W (Jeng et al., 2006), 175fs (Abel et al., 1999), C280G (Huang et al., 2005), S281X (Huang et al., 2005).

## Discussion

A key feature that emerges from the study of CMT1X is that Cx32 is a fundamental protein in the PNS as its dysfunction cannot be compensated by other mechanisms. In particular, loss of Cx32 in SCs of Cx32-null mice did not induce any compensatory change in the expression of other connexins (Nicholson et al., 2001). The expression of Cx32 in the PNS appears regulated by the transcription factors SOX10 and EGR2 which directly bind Cx32 promoter with synergistic action (Bondurand et al., 2001). Robust expression of glial fibrillary acidic protein (GFAP), the only non-myelinating SC marker persisting in adult Cx32-null mice, suggests that Cx32 is involved in regulating GFAP levels and coordinating the program of myelin gene expression (Nicholson et al., 2001). A recent microarray analysis of normal and injured sciatic nerves of WT and Cx32-null mice supports a crucial role for Cx32 in re-myelination of SCs (Freidin et al., 2015), both in normal axonal maintenance and regeneration following peripheral nerve injury, during which Cx32 is downregulated (Scherer et al., 1995). The same study, together with others (Kobsar et al., 2003; Groh et al., 2012; Klein et al., 2015), indicates that loss of Cx32 dysregulates several genes associated with immune response, thus contributing to the severity of the disease.

The identification of so-called “functional” CMT1X mutations, which retain the capacity to ensure normal electrical GJ coupling *in vitro*, suggests that permeability or gating abnormalities of Cx32 channels are *per se* sufficient to trigger a severe neuropathy. As Cx32 WT GJ channels are known to be permeable to Ca^2+^, cAMP, cGMP, IP_3_ and ATP (Harris, 2007), it is possible that these “functional” mutations alter second messenger or other cytoplasmic molecules signals, causing downregulation in the expression of genes that are required to maintain the myelinating state of SCs. In 1997, Oh et al. (Oh et al., 1997) hypothesized that the primary defect underlying CMT1X neuropathy in the presence of Cx32 mutants forming electrically conductive channels is the lower permeability of GJ channels to cAMP, which is involved in myelin homeostasis in SCs (LeBlanc et al., 1992). We have recently demonstrated that this theory is unlikely, at least with regard to the most studied CMT1X mutant (R220X; Carrer et al., 2018). Indeed, lack of Cx32 GJs in myelinating SCs does not appear to cause a slower radial diffusion of low molecular weight dyes along the myelin sheath of Cx32-null mouse with respect to the WT due to the presence of other connexins forming GJ channels (Balice-Gordon et al., 1998). Nonetheless, Cx32-null mice develop a late-onset peripheral neuropathy with demyelination features similar to those found in humans with Cx32 mutations (Nelles et al., 1996; Anzini et al., 1997; Scherer et al., 1998), indicating that other functions performed by Cx32 in SCs could be involved in the pathogenesis of the disease. The validity of the Cx32-null mouse as a model of Cx32 function has been strengthened by the demonstration that both transgenic and lentiviral expression of Cx32 in myelinating SCs ameliorates nerve pathology (Scherer et al., 2005; Sargiannidou et al., 2015), improving motor performance (Kagiava et al., 2016). The hypothesis that the key role of Cx32 in the myelination process does not involve GJ channels was initially suggested in Freidin et al. (2009) and supported by the recent observation that defective Cx32 GJ plaque formation in 14 CMT1X mutants correlates only with CNS abnormalities (Abrams et al., 2017).

**Figure 2:**
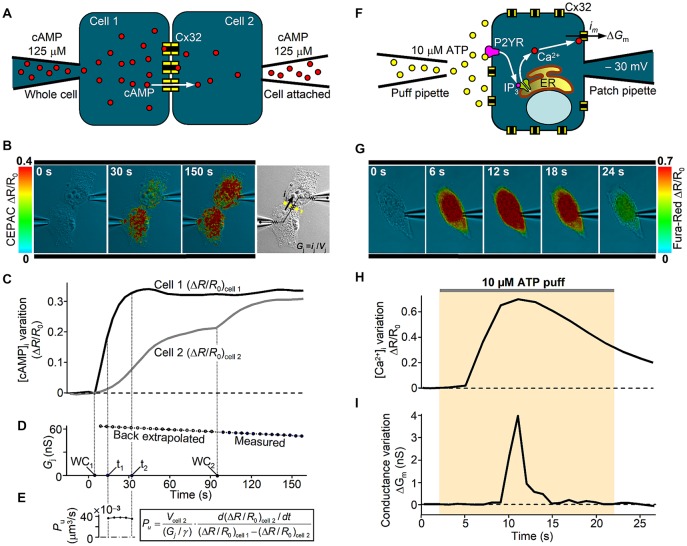
*In vitro* analysis of human Cx32 GJ and hemichannel functionality. **(A)** Scheme of dual patch clamp experiment to derive the unitary GJ permeability to cAMP in an isolated pair of Cx32-WT transfected HeLa cells, as in Carrer et al. (2018). At time zero, cAMP is injected in cell 1 under whole-cell recording conditions (WC1) and its intercellular transfer is monitored by FRET variation (Δ*R*/*R*_0_) of CEPAC sensor. **(B)** Three representative frames illustrate successive stages of an experiment. Around 90 s after WC1, the whole-cell configuration is achieved also in cell 2 (WC2), delivering the same concentration of cAMP and deriving the junctional conductance *G*_j_ from the current *i*_j_ elicited by a 10 mV voltage difference (*V*_j_) between the two pipettes, as illustrated in the bright field image. **(C,D)** Time course of the cell-averaged FRET signal and the junctional conductance *G*_j_ from the experiment in **(B)**. **(E)** A confocal z-stack was performed at the end of the experiment to derive cell 2 volume (*V*_cell 2_), which is required to compute the single channel permeability *P*_u_ to cAMP, as described in Hernandez et al. (2007), where γ is the single channel conductance. **(F)** Scheme of patch clamp experiment to study the hemichannel gating by [Ca^2+^]_i_ in a single Cx32-WT transfected HeLa cell. An IP_3_-dependent [Ca^2+^]_i_ transient is stimulated by an extracellular puff containing 10 μM ATP. Cx32 hemichannel opening and closure were monitored in terms of membrane conductance variation (ΔG_m_) computed by the periodic application (at 1 Hz) of a +10 μV voltage step lasting 100 ms. Contribution to ΔG_m_ by other Ca^2+^-activated channels was kept negligible by specific blockers contained in the extracellular solution. **(G)** Representative frame sequence of an experiment using Fura-Red ${\text{Ca}}_{\text{i}}^{2+}$ dye. **(H,I)** Time course from the experiment in **(G)** of the cell-averaged Fura-Red ΔR/R_0_ and the membrane conductance variation ΔG_m_ due to opening and closure of Cx32-WT hemichannels. For further details, see Carrer et al. (2018).

Despite the large amount of studies on mutant GJ properties, Cx32 hemichannel dysfunction was poorly investigated. Altered hemichannel gating properties could have devastating consequences for cellular function due to loss of ionic gradients and small metabolites and increased influx of Ca^2+^. A growing body of evidence also indicates that ATP-mediated paracrine signaling in SCs is critical for the myelination process, which supports the hypothesis that alteration of Cx32 hemichannel opening and ATP release could underlie CMT1X (Nualart-Marti et al., 2013; Carrer et al., 2018). Little is known about the physiological mechanism that controls opening and closure of Cx32 hemichannels in response to [Ca^2+^]_i_ changes (Cotrina et al., 2000; Belliveau et al., 2006; De Vuyst et al., 2006; Carrer et al., 2018), but we recently found that Cx32 hemichannels carrying the pathological C-terminus truncation R220X fail to open in response to a canonical IP_3_-mediated signal transduction cascade that elevates [Ca^2+^]_i_ (Carrer et al., 2018). A useful combination of patch-clamp and optical fluorescence microscopy for dissecting Cx32 channel properties is described in Figure 2. Interestingly, the gating function of R220X hemichannels was completely restored by both the intracellular and extracellular application of a 12 amino acid peptide that mimics the Cx32 cytoplasmic loop, suggesting that the C-terminal domain of Cx32 is not directly involved in the gating mechanism but acts as a modulator.

Molecular determinants of permeability/gating properties investigated in GJ channels and hemichannels of any connexin isoform, including Cx32, are still debated (Peracchia, 2004; Zhou et al., 2009; Oh and Bargiello, 2015), but their elucidation could help to answer why different Cx32 mutations cause a similar phenotype equivalent to a loss-of-function in the PNS of CMT1X patients. *In situ* investigation of signaling pathways mediated by Cx32 in pre-myelinating and myelinated SCs is also a pre-requisite for understanding how Cx32 dysfunction deregulates axon/SC homeostasis causing the CMT1X phenotype.

## Author Contributions

MB wrote the review article and prepared figures.

## Conflict of Interest Statement

The author declares that the research was conducted in the absence of any commercial or financial relationships that could be construed as a potential conflict of interest.
